# Acknowledgement to Reviewers of *Behavioral Sciences* in 2014

**DOI:** 10.3390/bs5010041

**Published:** 2015-01-08

**Authors:** Behavioral Sciences Editorial Office

**Affiliations:** MDPI AG, Klybeckstrasse 64, CH-4057 Basel, Switzerland

The editors of *Behavioral Sciences* would like to express their sincere gratitude to the following reviewers for assessing manuscripts in 2014:

Abejuela, Harmony Raylen
Agrillo, Christian
Allen, Andrew
Anderson, Kim
Arik, Engin
Bafna, Sonit
Baker, Dewleen
Balliet, Daniel
Barrett, Lisa
Barton, Kevin R.
Bauer, Dietmar
Bay, Esther
Beasley, Clare L.
Bedics, Jamie D.
Bendall, Sarah
Bienvenu, Joseph
Bohus, Martin
Bokde, Arun L.W.
Cacciola, John S.
Campbell-Arvai, Victoria
C’de Baca, Kathreen
Chan, Chiu-Shui
Chin, Seongah
Coverdale, John
Cowley, Stephen
Davenport, Nicholas D.
Davies, Bill
De Berardis, Domenico
Dean, Philip J. A.
Demarque, Christophe
Dolan, Sara
Dyer, Anne Sibilla
Edwards, Katie Sears
Elmore, Lauren Caitlin
Euser, Saskia
Fidalgo, C.
Ford, Julian D.
Frank, Ellen
Gabora, Liane
Gil, Jorge
Gómez Laplaza, Luis M.
Goodwyn, Erik
Gray, Whitney Austin
Hashimoto, Ryu-Ichiro
Heo, Yeonsook
Hidalgo, M. Carmen
Hirtle, Stephen C.
Hodgson, Tim
Horner, Michael D.
Isern, David
Jensen, Keith
Kalafatakis, Konstantinos
Kull, Kalevi
Lagro, James A.
Lane, Scott
Leliveld, Marijke
Lewicka, Maria
Lin, Yaw-jen
Logan, Alan C
Lu, Yi
Lysaker, Paul
Maller, Jerome J.
Matsuoka, Yutaka
McGurk, Susan
Meagher, Benjamin R.
Meilinger, Tobias
Melis, Alicia
Mendlowicz, Mauro Vitor
Mithoefer, Michael C.
Nickerson, Angela
Nurius, Paula
Ostwald, Michael
Pang, Ching Lin
Parra, Mario A.
Penn, Alan
Peterson, Alan L.
Pieterse, Alex L.
Rabinak, Christine A.
Ravizza, Susan M.
Reardon, Annemarie
Rhind, Daniel
Riva-Posse, Patricio
Rowland, Susan
Sadatsafavi, Hessam
Sammartino, Jonathan
Sawang, Sukanlaya
Schalinski, Inga
Schärer, Lars
Schnabel, Marc Aurel
Schuck, Nicolas W.
Smith, John
Snowden, Charles
Southworth, Michael
Taramp, Margaret
Teixeira, Carlos
Thompson, Deborah J.
Van Voorhees, Elizabeth E.
Villani, Daniela
Vonk, Jennifer
Wagner, Amy W.
Wahlberg, Lawrence
Walter, Kristen
Warneken, Felix
Werner, Nicole
Wicker, Allan
Wiener, Jan
Wilson, Debra Rose
Yamamoto, Shinya
Yoder, Matthew
Zandberg, Laurie
Zigman, Daniel
Zoladz, Phillip R.

